# Financial hardship and drug use among men who have sex with men

**DOI:** 10.1186/s13011-018-0159-0

**Published:** 2018-05-24

**Authors:** Su Hyun Park, Yazan Al-Ajlouni, Joseph J. Palamar, William C. Goedel, Anthony Estreet, Brian Elbel, Scott E. Sherman, Dustin T. Duncan

**Affiliations:** 1 Department of Population Health, New York University School of Medicine, 227 East 30th Street, 6th Floor, New York, NY 10016 USA; 2 Morgan State University School of Social Work, Baltimore, MD USA

**Keywords:** Financial hardship, Drug use, Tobacco, Alcohol, Men who have sex with men (MSM)

## Abstract

**Background:**

Little is known about the role of financial hardship as it relates to drug use, especially among men who have sex with men (MSM). As such, this study aimed to investigate potential associations between financial hardship status and drug use among MSM.

**Methods:**

We conducted a cross-sectional survey of 580 MSM in Paris recruited using a popular geosocial-networking smartphone application (GSN apps). Descriptive analyses and multivariate analyses were performed. A modified Poisson model was used to assess associations between financial hardship status and use of drugs (any drugs, tobacco, alcohol, marijuana, inhalant nitrites, and club drugs).

**Results:**

In our sample, 45.5% reported that it was somewhat, very, or extremely difficult to meet monthly payments of bills (high financial hardship). In multivariate analyses, a high level of financial hardship was significantly associated with an increased likelihood of reporting use of any substance use (adjusted risk ratio [aRR] = 1.15; 95% CI = 1.05–1.27), as well as use of tobacco (aRR = 1.45; 95% CI = 1.19–1.78), marijuana (aRR = 1.48; 95% CI =1.03–2.13), and inhalant nitrites (aRR = 1.24; 95% CI = 1.03–1.50).

**Conclusions:**

Financial hardship was associated with drug use among MSM, suggesting the need for interventions to reduce the burden of financial hardship in this population.

## Introduction

Gay, bisexual, and other men who have sex with men (MSM) are more likely to use illicit drugs compared to general population [1–5], perhaps given that they are more vulnerable to negative experiences in their daily lives. These experiences, described by Meyer’s minority stress model, include rejection, stigmatization, discrimination, and social isolation which is ultimately due to their sexual orientation [6, 7]. In 2016, data from a national, population-based sample from Australia showed that gay and bisexual men were significantly more likely than heterosexual men to use an illicit drug during their lifetime [8]. Additionally, in a longitudinal, community-based cohort comprising 13,519 US adolescents, gay males were shown to be at higher risk for concurrent polysubstance use than completely heterosexual individuals in repeated measures analyses [9]. Similarly, a study in the UK revealed that recreational drug use was greater among MSM; the rates of lifetime and past-month use of drugs, including mephedrone, ketamine, volatile nitrites, sildenafil, gamma hydroxybutyrate (GHB), and gamma-butyrolactone (GBL), were significantly higher in the MSM group than a non-MSM group [10]. Drug use, specifically among MSM, can motivate risky sexual behaviors, which in turn leads to negative health outcomes. In a cohort study from1998 to2008, Ostrow et al. [11] reported that a specific combination of sex-drugs contributed to the majority of HIV seroconversions among a sample of MSM (*n* = 1667) in the United States [11]. In addition, drug use can relate to poor mental health among MSM, including depressive symptoms [12].

Risk factors related to increased prevalence of drug use among MSM are multifaceted and complex. Previous research suggested that rejection, stigma, discrimination, and social isolation due to their sexual orientation are potential risk factors for drug use among MSM [13–15]. In addition to these factors, emerging research has explored the associations between financial hardship (when one has insufficient financial resources to adequately meet household needs) and drug use among sexual minority groups. Wong et al. [16] found that financial hardship was associated with illicit drug use in a sample of young MSM (*n* = 526) in Los Angeles, California. To the best of our knowledge, this is one of few studies conducted on financial hardship and drug use in MSM and this aforementioned study utilized experiences of childhood financial hardship as an indicator of socioeconomic status; thus, it does not necessarily represent recent financial hardship status. While evidence for a relationship between financial hardship and drug use remains scant, MSM groups are more likely to experience financial hardships [17, 18], which may be associated with increased likelihood of drug use. MSM individuals suffer from an average wage penalty of approximately − 6.5% when compared to heterosexual men in France [19]. Furthermore, it is important to note that, despite the economic growth in Western Europe and France, gaps in income inequality have widened and the unemployment rate in France is estimated to be above 10% [20, 21].

The objective of this study was to examine the association between financial hardship and drug use among a sample of gay, bisexual, and other MSM in the Paris (France) metropolitan area who were recruited from a popular geosocial networking application for MSM. We focus on MSM in France because gaps in income inequality have widened and the unemployment rate in France is approximated to be above 10% [20, 22]. Such an increase in income inequality suggests that MSM who were previously experiencing financial hardship may continue to do so.

## Methods

### Study participants

For this study, a popular geosocial networking smartphone application (app) for MSM was used to recruit participants by means of a broadcast advertisement. The advertisements were limited to users in Paris (France) metropolitan area. Consistent with previous studies [23], users were shown an advertisement with text encouraging them to click through the advertisement to complete an anonymous web-based survey. To encourage participation, the advertisement stated that users who completed the survey would have a chance of winning €65 (approximately $70). Upon clicking the advertisement, users were directed to a landing page where they provided informed consent and initiated a 52-item online survey.

Details of the study design and methods have been presented previously [24]. Briefly, the survey was offered in both French and English. The survey was translated by three native French speakers, and subsequently reviewed and adjudicated by a fourth native French speaker. A fifth French speaker and health researcher pretested and finalized the survey by back-translation. The majority of respondents (94.3%) took the survey in French, and the survey took an average 11.4 min (SD = 4.0) for users to complete. Among 5206 users who clicked on the advertisement and reached the landing page of the survey, 935 users provided informed consent and began the survey, and 580 users signed informed consent and completed the survey. Thus, the overall response rate was 11.1% and the completion rate of 62.0%. The protocols were approved by the New York University School of Medicine Institutional Review Board before data collection. All participants reported being at least 18 years old at the time of survey administration.

### Financial hardship

Financial hardship was measured using the previously reported question [25, 26], “How difficult is it for you to meet monthly payments on bills?” Response options included: “not at all difficult”; “not very difficult”; “somewhat difficult”; “very difficult”; and “extremely difficult”. The following binary variable was created: high financial hardship (somewhat difficult; very difficult; and extremely difficult) and low financial hardship (not at all difficult and not very difficult), consistent with prior research [26]. A trichotomous measure of financial hardship was also analyzed: high (“very difficult” and “extremely difficult”), medium (“somewhat”) and low (“not at all difficult” and “not very difficult”).

### Tobacco, alcohol and drug use

Participants were asked about their use of drugs during the prior 3 months. The substances included were cigarettes, electronic cigarettes or nicotine vapes, alcohol (five or more drinks in one sitting), marijuana, synthetic cannabinoids (“synthetic marijuana”), cocaine, ecstasy (3,4-methylenedioxymethamphetamine [MDMA]), ketamine, GHB and GBL, methamphetamine, heroin, prescription stimulants, prescription benzodiazepines, inhalant nitrites, other inhalants (e.g., glue, solvents, and gas), non-medical use of prescription opioids, psychedelics (e.g., lysergic acid diethylamide [LSD] and psilocybin mushrooms), new psychedelics (e.g., psychedelic phenethylamines and *N*,*N*-dimethyltryptamine [DMT]), synthetic cathinones (e.g., bath salts), and anabolic steroids. For analytic purposes, composite variables were created. Overall use was defined as the use of any substance described above. Any drug use was defined as the use of any product except tobacco (cigarettes and e-cigarettes) use and alcohol. Tobacco use included traditional cigarettes and electronic cigarettes (nicotine vapes). Club drugs included ecstasy (MDMA), ketamine, GHB and GBL. Alcohol, marijuana and inhalant nitrite use were also included in the analyses as separate, distinct variables.

### Socio-demographic covariates

The following socio-demographic covariates were included: age (18–24, 25–29, 30–39, 40–49, or ≥ 50 years), born in France (yes, no), sexual orientation (gay, bisexual), employment status (employed, unemployed, student), and current relationship status (single, relationship with a man).

### Statistical analyses

Data were analyzed using descriptive statistics by drug use. Multivariate analyses were conducted to examine the association between financial hardship status and use of drugs (any drug, tobacco, alcohol, marijuana, inhalant nitrites, and club drugs) after adjustment for socio-demographic covariates. The modified Poisson model (generalized linear models [GLMs] using Poisson and log link), suggested by Zou [27] used to calculate the adjusted relative risks (aRRs) and corresponding 95% confidence intervals (CI) due to the convergence issues of the log-binomial model. Data analysis was performed using Stata version 14.0 (StataCorp, College Station, TX). A two-sided *p*-value < 0.05 was considered to indicate statistical significance.

## Results

The socio-demographic and financial hardship characteristics of the study participants are shown in Table 1. Of the 580 MSM, the mean age of the sample was 35.24 ± 9.94 years with a median of 35 years (range: 18–66 years). 63% were less than 40 years old. More than 65% of the participants reported that they were employed and not currently in a relationship (e.g., single). More than 45% reported that they found it somewhat, very, or extremely difficult to meet monthly payments of bills. Among the sample, 43.1% reported that they have used cigarettes or e-cigarettes, 19.5% have used marijuana, and 46.7% have used inhalant nitrites. Among those who reported a high level of financial hardship, 83.7% reported that they have used any type of products, 54.5% reported that they have used any type of drugs, 53.4% reported that they have used inhalant nitrites, and 52.7% reported that they have used tobacco in the past three months.

**Table 1:** Financial hardship and drug use among men who have sex with men in Paris (*N* = 580)

	Total	Overall use^a^	Any drug^b^	Tobacco use^c^	Alcohol use	Marijuana use	Inhalant nitrites use	Club drug use^d^
Total	580 (100)	438 (75.5)	316 (54.5)	250 (43.1)	271 (46.7)	113 (19.5)	271 (46.7)	68 (11.7)
Age								
18–24	84 (14.5)	70 (83.3)	40 (47.6)	45 (53.6)	55 (65.5)	23 (27.4)	28 (33.3)	9 (10.7)
25–29	103 (17.8)	83 (80.6)	69 (67.0)	52 (50.5)	60 (58.3)	35 (34.0)	59 (57.3)	20 (19.4)
30–39	180 (31.0)	137 (76.1)	99 (55.0)	76 (42.2)	87 (48.3)	26 (14.4)	89 (49.4)	25 (13.9)
40–49	139 (24.0)	104 (74.8)	73 (52.5)	58 (41.7)	50 (36.0)	24 (17.3)	65 (46.8)	10 (7.2)
≥ 50	54 (9.3)	37 (68.5)	29 (53.7)	16 (29.6)	17 (31.5)	5 (9.3)	24 (44.4)	4 (7.4)
Sexual orientation								
Gay	487 (84.0)	377 (77.4)	276 (56.7)	216 (44.4)	237 (48.7)	101 (20.7)	236 (48.5)	64 (13.1)
Bisexual	69 (11.9)	48 (69.6)	30 (43.5)	26 (37.7)	27 (39.1)	9 (13.0)	25 (36.2)	2 (2.9)
Born in France								
Yes	450 (77.6)	353 (78.4)	240 (55.6)	200 (44.4)	224 (49.8)	86 (19.1)	217 (48.2)	58 (12.9)
No	113 (19.5)	80 (70.8)	62 (54.9)	47 (41.6)	45 (39.8)	27 (23.9)	50 (44.3)	10 (8.9)
Employment status								
Employed	388 (66.9)	291 (75.0)	208 (53.6)	165 (42.5)	178 (45.9)	61 (15.7)	186 (47.9)	48 (12.4)
Unemployed	84 (14.5)	71 (84.5)	57 (67.9)	36 (42.9)	41 (48.8)	21 (25.0)	49 (58.3)	10 (11.9)
Student	81 (14.0)	65 (80.3)	43 (53.1)	44 (54.3)	48 (59.3)	31 (38.3)	28 (34.6)	10 (12.4)
Current Relationship								
Single	378 (65.2)	288 (76.2)	201 (53.2)	162 (42.9)	186 (49.2)	76 (20.1)	168 (44.4)	47 (12.4)
Relationship with a man	172 (29.7)	136 (79.1)	105 (61.1)	77 (44.8)	78 (45.4)	35 (20.4)	94 (54.7)	21 (12.2)
Financial Hardship								
Low	297 (51.2)	211 (71.0)	144 (48.5)	108 (36.4)	134 (45.1)	46 (15.5)	125 (42.1)	30 (10.1)
High	264 (45.5)	221 (83.7)	167 (63.3)	139 (52.7)	135 (51.1)	67 (25.4)	141 (53.4)	38 (14.4)

Values are presented as n(%)

^a^Includes cigarettes, electronic cigarettes or nicotine vapes, alcohol, marijuana, synthetic marijuana, cocaine, MDMA, ketamine, GHB/GBL, methamphetamine, heroin, prescription stimulants, prescription benzodiazepines, inhalant nitrites, other inhalants, prescription opioids, psychedelics, synthetic cathinones, and anabolic steroids

^b^Includes marijuana, synthetic marijuana, cocaine, MDMA, ketamine, GHB/GBL, methamphetamine, heroin, prescription stimulants, prescription benzodiazepines, inhalant nitrites, other inhalants, prescription opioids, psychedelics, synthetic cathinones, and anabolic steroids

^c^Includes cigarettes and electronic cigarettes (nicotine vapes)

^d^Includes MDMA, ketamine, and GHB/GBL

The multivariate associations of financial hardship and drug use are presented in Table 2. Reporting of a high level of financial hardship was associated with an increased risk of use of any type of products (aRR = 1.15; 95% CI = 1.05–1.27), as well as use of any type of drugs (aRR = 1.25; 95% CI = 1.07–1.46), tobacco (aRR = 1.45; 95% CI = 1.19–1.78), marijuana (aRR = 1.48; 95% CI = 1.03–2.13), and inhalant nitrites (aRR = 1.24; 95% CI = 1.03–1.50). When using the trichotomous variable of financial hardship, similar associations with greater risk ratios were observed (p-trend< 0.05 for overall, tobacco, marijuana and inhalant nitrites use). No association between financial hardship, alcohol use and club drug use was observed.

**Table 2:** Adjusted risk ratios (aRRs)^a^ for the association between financial hardship and drug use

	Overall use^b^	Any Drug^c^	Tobacco use^d^	Alcohol use	Marijuana use	Inhalant Nitrites use	Club drug use^e^
	aRR (95% CI)	aRR (95% CI)	aRR (95% CI)	aRR (95% CI)	aRR (95% CI)	aRR (95% CI)	aRR (95% CI)
Financial hardship							
Model 1							
Low	Referent	Referent	Referent	Referent	Referent	Referent	Referent
High	1.15 (1.05, 1.27)**	1.25 (1.07, 1.46)**	1.45 (1.19, 1.78)**	1.10 (0.93, 1.32)	1.48 (1.03, 2.13)*	1.24 (1.03, 1.50)*	1.47 (0.91, 2.37)
Model 2							
Low	Referent	Referent	Referent	Referent	Referent	Referent	Referent
Medium	1.14 (1.03, 1.26)*	1.21 (1.02, 1.43)*	1.37 (1.10, 1.71)**	1.14 (0.95, 1.37)	1.37 (0.92, 2.03)	1.18 (0.96, 1.45)	1.46 (0.87, 2.44)
High	1.19 (1.06, 1.34)**	1.37 (1.12, 1.67)**	1.67 (1.30, 2.16)**	1.01 (0.77, 1.32)	1.80 (1.13, 2.85)*	1.40 (1.11, 1.78)**	1.48 (0.75, 2.92)
Test for trend	*p* = 0.002	*p* = 0.003	*p* < 0.0001	*p* = 0.397	*p* = 0.013	p = 0.01	*p* = 0.124

*aRR* adjusted risk ratio, *CI* Confidence Intervals

^a^Adjusted for age, sexual orientation, origin (born in France), employment and relationship status

^b^Includes cigarettes, electronic cigarettes or nicotine vapes, alcohol, marijuana, synthetic marijuana, cocaine, MDMA, ketamine, GHB/GBL, methamphetamine, heroin, prescription stimulants, prescription benzodiazepines, inhalant nitrites, other inhalants, prescription opioids, psychedelics, synthetic cathinones, and anabolic steroids

^c^Includes marijuana, synthetic marijuana, cocaine, MDMA, ketamine, GHB/GBL, methamphetamine, heroin, prescription stimulants, prescription benzodiazepines, inhalant nitrites, other inhalants, prescription opioids, psychedelics, synthetic cathinones, and anabolic steroids

^d^Includes cigarettes and electronic cigarettes (nicotine vapes)

^e^Includes MDMA, ketamine and GHB/GBL

Model 1: high financial hardship (Somewhat difficult; Very difficult; and Extremely difficult) and low financial hardship (Not at all difficult and Not very difficult)

Model 2: high financial hardship (very difficult and extremely difficult), somewhat difficult, and low (not at all and not very difficult)

*p < 0.05; **p < 0.01

## Discussions

To the best of our knowledge, this is the first study to have examined the association between financial hardship and drug use among MSM in the European Union, as well as one of few studies examining the association between recent financial hardship and drug use among any MSM population. The results demonstrate that almost half of the participants had experienced financial hardship (46%) and the majority (83.7%) of them had used at least one type of drug in the past 3 months. In addition to alcohol use, inhaled nitrites was the most commonly reported drug used in this sample, consistent with other studies among MSM [28, 29]. Although the effect sizes were of relatively small magnitude, our findings suggest that higher levels of financial hardship are significantly associated with overall drug use, as well as the use of tobacco, marijuana and inhaled nitrites after adjusting for covariates. This result is meaningful as effect sizes can be affected by sample characteristics and should be interpreted in a research-specific context [30, 31]. Wong et al. [16] presents similar result reporting that childhood financial hardship was associated with increased risk of recent drug use among young MSM in Los Angeles [16]. However, no significant associations were observed between alcohol and club drug use in this study.

Meyer [6] proposed the minority stress perspective [6] to conceptualize the association between increased levels of stress due to exposure to victimization, discrimination, rejection, hostility and negative attitudes about homosexuality, and greater levels of drug use among sexual minority populations. Since financial hardship could be both a cause and a consequence of discrimination [32], there is a possibility that experiencing financial hardship may contribute to this type of stress, which may result in increased drug use among MSM.

Furthermore, there were several limitations to this study that are important to mention. First, there was a difference between the exposure and outcome measurements with regard to the time period included; financial hardship was measured at the time of the survey, while drug use was measured based on the past 3 months. Therefore, participants may have reported financial hardship during any period of their lives, including current or past hardships, or hardships spanning the lifetime. In addition, no causal inferences can be drawn due to the cross-sectional design of the study. Reverse causality and a potential bidirectional relationship cannot be ruled out (e.g., consumption of drugs may contribute to financial hardship). Also, our study relies on a single item to measure of financial hardship as conducted by other previous studies [26, 33]. Future studies involving multiple scales/indicators of financial hardship are warranted. Moreover, self-reporting was used to collect data, which could have introduced social desirability bias, reporting bias, or recall bias, particularly among MSM [34]. Therefore, for example, we may have underestimated the exact prevalence of drug use. Because some of study variables were not collected with the aim of maximizing the participation rate, there may have been residual confounding from other unmeasured covariates (e.g., income, education status, race/ethnicity and binge drinking) related to substance use. Finally, we focused on MSM in the Paris metropolitan area who used a single geo-social networking application. The relatively low response rate precludes generalization of our results.

Despite these limitations, this study adds to the body of literature, highlighting general drug use in addition to specific drug use, and describes the association of financial hardship and drug use among MSM. Future research with MSM should utilize longitudinal and qualitative research to better understand causal relationships and identify mechanisms for the association found. This research can provide better direction of structural interventions and policies to reduce health inequalities, identify factors (e.g., social exclusion, discrimination) associated with financial hardship and provide drug screening services to MSM.

## Conclusions

We found that financial hardship was associated with overall drug use, tobacco, marijuana and inhaled nitrites among a sample of MSM in the Paris metropolitan area. Future studies should investigate the causal pathways that may link financial hardship to drug use. Our findings will be of value in developing effective prevention measures that address drug use among MSMs.

## Abbreviations

CI: Confidence Interval
DMT: *N*,*N*-dimethyltryptamine
GBL: Gamma-butyrolactone
GHB: Gamma hydroxybutyrate
GLM: Generalized Linear Model
LSD: Lysergic acid diethylamide
MDMA: 3,4-methylenedioxymethamphetamine
MSM: Men who have sex with men
RR: Relative Risk
